# The *Aspergillus flavus* Phosphatase CDC14 Regulates Development, Aflatoxin Biosynthesis and Pathogenicity

**DOI:** 10.3389/fcimb.2018.00141

**Published:** 2018-05-07

**Authors:** Guang Yang, Yule Hu, Opemipo E. Fasoyin, Yuewei Yue, Lijie Chen, Yue Qiu, Xiuna Wang, Zhenhong Zhuang, Shihua Wang

**Affiliations:** Key Laboratory of Pathogenic Fungi and Mycotoxins of Fujian Province, Key Laboratory of Biopesticide and Chemical Biology of Education Ministry, School of Life Sciences, Fujian Agriculture and Forestry University, Fuzhou, China

**Keywords:** *Aspergillus flavus*, phosphatase, *AflCDC14*, aflatoxin, pathogenicity

## Abstract

Reversible protein phosphorylation is known to play important roles in the regulation of various cellular processes in eukaryotes. Phosphatase-mediated dephosphorylation are integral components of cellular signal pathways by counteracting the phosphorylation action of kinases. In this study, we characterized the functions of CDC14, a dual-specificity phosphatase in the development, secondary metabolism and crop infection of *Aspergillus flavus*. Deletion of *AflCDC14* resulted in a growth defect and abnormal conidium morphology. Inactivation of *AflCDC14* caused defective septum and failure to generate sclerotia. Additionally, the *AflCDC14* deletion mutant (Δ*CDC14*) displayed increased sensitivity to osmotic and cell wall integrity stresses. Importantly, it had a significant increase in aflatoxin production, which was consistent with the up-regulation of the expression levels of aflatoxin biosynthesis related genes in Δ*CDC14* mutant. Furthermore, seeds infection assays suggested that *AflCDC14* was crucial for virulence of *A. flavus*. It was also found that the activity of amylase was decreased in Δ*CDC14* mutant. AflCDC14-eRFP mainly localized to the cytoplasm and vesicles during coidial germination and mycelial development stages. Taken together, these results not only reveal the importance of the CDC14 phosphatase in the regulation of development, aflatoxin biosynthesis and virulence in *A. flavus*, but may also provide a potential target for controlling crop infections of this fungal pathogen.

## Introduction

*Aspergillus flavus* is a saprophytic and pathogenic fungus which contaminates a variety of economical crops (such as peanuts and maize) with mycotoxins, causing huge economic losses (Amaike and Keller, 2011; Bhatnagar-Mathur et al., 2015; Lim et al., 2015). In addition, this fungus is also an opportunistic pathogen capable of causing aspergillosis or liver cancer in immunocompromised mammalian hosts (Hedayati et al., 2007). Aflatoxins (AFs) are a major mycotoxin mainly produced by *A. flavus* and *A. parasiticus*, and AFs are the most toxic, deleterious and carcinogenic secondary metabolites of fungi, posing a serious threat to animals and humans (Yang et al., 2015; Han et al., 2016). Chronic exposure to low concentrations of aflatoxins may lead to immunosuppression, growth impairment and liver cancer (Khlangwiset et al., 2011). Previous studies have elucidated the gene cluster of aflatoxin biosynthesis (Yabe and Nakajima, 2004), but the regulation of aflatoxin biosynthesis has not been identified.

In recent years, post-translational modification (PTM) which includes phosphorylation (Bodenmiller et al., 2010; Shwab et al., 2017), methylation (McBride et al., 2007; Wang et al., 2012), acetylation (Xiong et al., 2010; Zhang et al., 2017), and SUMOylation (Castro et al., 2015; Nie et al., 2016), have been demonstrated to play important roles in various biological processes. In eukaryotic organisms, phosphorylation and dephosphorylation, which are regulated by protein kinases and phosphatases respectively, mainly occur on three animo acids including serine, threonine and tyrosine, and a balance of phosphorylation and dephosphorylation is required for the coordination of diverse biological events (Turrà et al., 2014; Yun et al., 2015). It has been proposed that kinase-mediated phosphorylation is involved in cell differentiation, secondary metabolism and virulence in filamentous fungi. In plant pathogenic fungi *F. graminearum*, cyclin-dependent kinases (CDKs) which are related to cell cycle are necessary to regulate growth and development (Liu et al., 2015). In human pathogen *C. albicans* (Wilson and Hube, 2010) and corn smut fungus *U. maydis* (Pérezmartín et al., 2006), it has also been suggested that CDKsare required for cell cycle progression in morphology and virulence. These findings indicate that proper phosphorylation in cell cycle process may be crucial for the development and pathogenicity of filamentous fungi. In contrast to the numerous studies of kinases in different fungi, there have been only few studies of phosphatase regulating cell cycle in filamentous fungi.

As a dual-specificity phosphatase which removes the phosphotryosine and phosphoserine/theronine residues, CDC14 is highly conserved in almost all eukaryotes (Mocciaro and Schiebel, 2010). CDC14 is known mostly for its role of regulating mitosis, especially in late M phase (Kao et al., 2014). As studied in budding yeast *S. cerevisiae*, CDC14 is required for mitotic exit and cytokinesis by triggering the inactivation of cell cycle associated CDKs at the end of mitosis (Yuste-Rojas and Cross, 2000; Miller et al., 2015). Moreover, CDC14 may participate in multi-stress responses, including osmotic, cell wall integrity and oxidative stress (Saito and Tatebayashi, 2004; Breitkreutz et al., 2010). In the fission yeast *S. pombe, CDC14* homolog *Clp1* was shown to be involved in coordinating cytokinesis, in collaboration with septation initiation network (SIN) (Trautmann et al., 2001; Trautmann and Mccollum, 2005). In plant-pathogenic fungi *F. graminearum* (Li et al., 2015) and *M. oryzae* (Li et al., 2016), deletion of *CDC14* gene resulted in defective phenotype, septum and virulence, indicating that *CDC14* is necessary for cell separation and morphogenesis. Additionally, inactivation of *CDC14* in *B. bassiana* severely affected vegetative growth, multi-stress response and virulence (Wang et al., 2013). In *C. albicans, CaCDC14* is not essential for vegetative growth, but it is important for asexual development and cell division (Clemente-Blanco et al., 2006). In *Aspergillus spp*, the *A. nidulans CDC14* null mutant led to a reduction of conidiation and secondary metabolite biosynthesis (Son and Osmani, 2009).

Despite the various roles played by *CDC14* orthologs in different cellular processes, the function of *CDC14* in *A. flavus* has not been characterized. In this study, we generated a *CDC14* gene deletion mutant, and analyzed the multiple phenotype, virulence and secondary metabolism in *A. flavus*. Our results suggest that *AflCDC14* may play an important role in asexual development, sclerotial formation, pathogenicity, stress response and secondary metabolism in *A. flavus*, and may be used as a potential target for curbing the threats posed by *A. flavus*.

## Materials and methods

### Strain and culture conditions

*Aspergillus flavus* strains used in this study were listed in Table 1. *A. flavus CA14* PTS was used as the parental strain for transformation. The wild type (WT) and the mutants generated in this study were grown on yeast extract-sucrose agar (YES), potato dextrose agar (PDA) and yeast extract-glucose agar (YGT) for mycelial growth and conidiation assays, and in Wickerham's medium (WKM) for sclerotia production at 37°C (Yang et al., 2016a). YES liquid medium and Potato dextrose broth (PDB) were used to detect aflatoxin production at 29°C (Yang et al., 2016b). All experiments were repeated at least three times.

**Table 1:** *Aspergillus flavus* strains used in this study.

**Strain**	**Genotype**	**Description**
*A. flavus CA14* PTS	Δ*ku70*, Δ*pyrG*	Chang et al., 2010
*A. flavus* wild-type	Δ*ku70*, Δ*pyrG*::*AfpyrG*	This study
*A. flavus* Δ*CDC14*	Δ*ku70*, Δ*CDC14*::*AfpyrG*	This study
*A. flavus* Δ*CDC14-*Com	Δ*ku70*, Δ*CDC14*:: *AfpyrG, CDC14*(p)::*CDC14*::*ptrA*	This study
*A. flavus CDC14-eRFP*	Δ*ku70, CDC14*(p)::*CDC14*-*eRFP*::*AfpyrG*	This study

### Targeted gene deletion and complementation of the *CDC14* gene

To generate the *CDC14* deletion strain (Δ*CDC14*) and the Δ*CDC14* complementary strain (Δ*CDC14*-Com) of *A. flavus*, protoplast preparation and transformation experiments were performed using previously described protocols (Chang et al., 2010). Primers used in this study were listed in Table 2. The upstream and downstream fragments of *CDC14* gene were amplified from genomic DNA of *A. flavus* WT strain with primer pairs *CDC14-*p1/p3 and *CDC14-*p4/p6, respectively. The *pyrG* selectable marker was amplified from *A. fumigatus* genomic DNA with primer pair *pyrG-*F/R. A fusion PCR strategy was used to generate the *CDC14* overlap PCR product, and then the overlap product was transformed into protoplasts of *A. flavus CA14* PTS strain. For complementation, a 3.8 kb PCR product including a 1.8 kb *CDC14* coding region and 2 kb promoter region was amplified using primers *CDC14*-com-F/R from the genomic DNA of *A. flavus* WT strain, and then cloned into the digested pPTRI vector using T4 DNA ligase (Takara). The recombinant pPTR-*CDC14* was transformed into protoplasts of the Δ*CDC14* mutant with pyrithiamine selectable marker. The mutants were verified by PCR, reverse transcription PCR (RT-PCR), and further confirmed by Southern blot analysis.

**Table 2:** Primers used for gene deletion and complementation.

**Primers**	**Sequence (5′/ 3′)**	**Application**
*CDC14*-p1	GGTCATTGCCCGCAGATT	CDC14 deletion
*CDC14*-p3	GGGTGAAGAGCATTGTTTGAGGCGGGATCGAGGCGACCTA	
*CDC14*-p4	GCATCAGTGCCTCCTCTCAGACATGTGCCTCCTACTACCC	
*CDC14*-p6	AAGTCCGAATGAACCTCA	
*PyrG-*F	GCCTCAAACAATGCTCTTCACCC	
*PyrG-*R	GTCTGAGAGGAGGCACTGATGC	
*CDC14*-p2	TCATTGCCCGCAGATTAC	
*CDC14*-p5	ATGGGCAGGTATCTCACG	
*CDC14*-OR	TCCCTTATCCTTCCGAGCAA	Mutant screen
*CDC14*-OF	TGGTCAATGTTGCCGAGT	
P801	CAGGAGTTCTCGGGTTGTCG	
P1020	CAGAGTATGCGGCAAGTCA	
*CDC14*-Com-F	GACCATGATTACGCCAAGCTTAGACACGAGGGAGACAGT	*CDC14*
*CDC14*-Com-R	GAATTCGAGCTCGGTACCCGGGGGGTAGTAGGAGGCAC	Complementation
*CDC14-eRFP-*OR	GACTTCGGTCCACTCCAC	*CDC14-eRFP*
*CDC14-eRFP-*OF	CTCGCCCTTGCTCACCATTTTCACACGAGTCGGGCTGC	construct
*eRFP-*F	ATGGTGAGCAAGGGCGAG	
*eRFP-*R	GGGTGAAGAGCATTGTTTGAGGCCTACTTGTACAGCTCGT	
*CDC14-*BF	GCATCAGTGCCTCCTCTCAGACGTTGCTTCTGCTGGACTG	
*CDC14-*BR	ACTGTCTCCAGGCAGCCCAC	
*CDC14-eRFP*-2	CAGGCTGACCCTCCTTAT	
*CDC14-eRFP*-5	CATACCAATCAACCCACC	

### Mycelial growth, conidiation, and sclerotia analysis

The phenotypes of all strains (WT, Δ*CDC14*, Δ*CDC14*-Com) were observed using different media. To assess the colony morphology and mycelial growth, about 10^4^ spores of each strain were point-inoculated onto YES, PDA, YGT, and GMM agar medium, respectively, and then cultured at 37°C for 5 days in the dark. Colony diameters were measured daily. For quantitative comparison of conidia, conidia were collected with 7% DMSO and 0.5% Tween-20 from PDA and YGT agar plates. The spores were counted using a hemocytometer and microscope. For sclerotial formation analysis, each strain was inoculated and grown on WKM agar medium at 37°C in the dark for 7 days. Then, 70% ethanol was used to wash away mycelia and conidia on the surface of the medium. Each experiment was performed thrice with four replicates.

### Aflatoxins analysis

To determine aflatoxin production, a procedure of thin layer chromatography (TLC) was used as previously described (Yang et al., 2016a). Fifteen milliliter of liquid YES or PDB medium was inoculated with 1 mL spore suspension (10^6^ spores/mL), and cultures were incubated at 29°C in the dark for 6 days. AF was extracted from the media as previously described (Yang et al., 2016a). The AF extraction samples were identified by thin layer chromatography (TLC) in a solvent system (chloroform: acetone = 9:1), and the plates were examined under UV light at 365 nm. Then, Gene Tools software was used for quantitative analysis of the AF produced.

### Stress assay

To determine the role of *AflCDC14* gene in *A. flavus* response to various stresses, all strains were point-inoculated onto PDA medium supplemented with the following agents: osmotic stress agents (1 M NaCl and 1 M KCl), cell wall stress agents (200 μg/mL CFW-calcofluor white and 200 μg/mL CR-Congo red), oxidative stress agent (5 mM H_2_O_2_) and genome integrity stress agent (0.01% MMS). After 5 days incubation at 37°C in the dark, the relative inhibition rates were calculated. The assays were repeated at least three times.

### Seeds infection assays

Pathogenicity assays on crop seeds were conducted as described previously (Kale et al., 2008). The pathogenicity of *A. flavus* is reported to be judged via conidia production and growth ability on crop seeds (Yang et al., 2016b). Conidia of all *A. flavus* strains were inoculated onto sterilized peanut and maize seeds. After incubation at 29°C for 6 days in the dark, the seeds were harvested in 50 mL centrifuge tubes with 15 mL sterile water supplemented with 0.05% Tween 20 for conidia quantification and aflatoxin assays, and vortexed for 2 min to mix the spores on the surface of seeds. The amount of conidia were calculated using a hemocytometer and microscope, and AF produced were quantified as previously mentioned in aflatoxin analysis.

### Subcellular localization

*A. flavus CDC14-eRFP* strain was used for protein localization according to the former approach (Yang et al., 2017). To generate the *CDC14-eRFP* fusion construct, four individual fragments were amplified by PCR. Briefly, the *CDC14* open reading frame (ORF) without the termination codon (TAA), and the eRFP fragment were amplified using primers pairs *CDC14-eRFP-*OR/OF and *eRFP*-F/R, respectively. Primers pairs *CDC14-eRFP*-BF/BR and *pyrG-*F/R were used to amplify the 1.5 kb downstream fragment and the selectable marker *pyrG*, respectively. The above four fragments were fused by overlap PCR as described before and transformed into protoplasts of *A. flavus CA14* PTS strain. After verification of *CDC14-eRFP* strain, 12 and 24 h growth mycelia were harvested and used to analyze the subcellular localization of *CDC14-eRFP* strain by using a Leica SP8 microscope. The vesicle of conidia and mycelia were stained with chloromethyl derivative of aminocoumarin (CMAC) for 1 h (Castro et al., 2016), and dual-channel imaging was used to observe the subcellular localization of *CDC14-eRFP* as described previously.

### Quantitative RT-PCR

The gene expression level was assessed by qRT-PCR (quantitative reverse transcription PCR). Mycelia collected from 48 h PDA and WKM cultures of WT and all mutant strains were used for total RNA isolation with TRIzol reagent (Biomarker Technologies, Beijing, China), and then cDNA was synthesized with First-Strand cDNA Synthesis Kit (TransGen Biotech, Beijing, China). cDNA was used as template for qRT-PCR analysis with SYBR Green qPCR mix (TaKaRa Biotechnology, Japan) in PikoReal Real-time PCR system (Thermo Fisher Scientific, USA). The related primers were listed in Table 3. The relative transcript level of each gene was calculated by the 2^−ΔΔCt^ method (Livak and Schmittgen, 2001), and *actin* was used as endogenous standard. All experiments were carried out in triplicate.

**Table 3:** Primers used for qRT-PCR.

**Primers**	**Sequence (5′/ 3′)**	**Application**
*brlA*-F	GCCTCCAGCGTCAACCTTC	*brlA* qRT-PCR
*brlA*-R	TCTCTTCAAATGCTCTTGCCTC	
*abaA*-F	TCTTCGGTTGATGGATGATTTC	*abaA* qRT-PCR
*abaA*-R	CCGTTGGGAGGCTGGGT	
*nsdC*-F	GCCAGACTTGCCAATCAC	*nsdC* qRT-PCR
*nsdC*-R	CATCCACCTTGCCCTTTA	
*nsdD*-F	GGACTTGCGGGTCGTGCTA	*nsdD* qRT-PCR
*nsdD*-R	AGAACGCTGGGTCTGGTGC	
*CDC15-*F	ACAACCTGGAGACTCGGATC	*CDC15* qRT-PCR
*CDC15-*R	AGGGTTCTGTGCTAGGATGG	
*TAO3-*F	CCACCTCCACCGGATATCAA	*TAO3* qRT-PCR
*TAO3-*R	TGCTCTTGTACGGTGAGTGT	
*aflR-*F	AAAGCACCCTGTCTTCCCTAAC	*aflR* qRT-PCR
*aflR-*R	GAAGAGGTGGGTCAGTGTTTGTAG	
*aflS-*F	CGAGTCGCTCAGGCGCTCAA	*aflS* qRT-PCR
*aflS-*R	GCTCAGACTGACCGCCGCTC	
*aflC-*F	GTGGTGGTTGCCAATGCG	*aflC* qRT-PCR
*aflC-*R	CTGAAACAGTAGGACGGGAGC	
*aflD-*F	GTGGTGGTTGCCAATGCG	*aflD* qRT-PCR
*aflD-*R	CTGAAACAGTAGGACGGGAGC	
*aflK-F*	GAGCGACAGGAGTAACCGTAAG	*aflK* qRT-PCR
*aflK-R*	CCGATTCCAGACACCATTAGCA	
*aflQ-F*	GTCGCATATGCCCCGGTCGG	*aflQ* qRT-PCR
*aflQ-R*	GGCAACCAGTCGGGTTCCGG	
*actin-F*	ACGGTGTCGTCACAAACTGG	The endogenous gene
*actin-R*	CGGTTGGACTTAGGGTTGATAG	

### Statistical analysis

All data were presented as the means ± standard deviation (SD) of three biological replicates samples. Graph Pad Prism 5 software was used for statistical and significance analysis, and recognized significance if *p*-values were < 0.05. Student's *t*-test was used to compare two means. Results from various assays were differentiated among the tested strains by one-way analysis of variance. Error bars represent standard error for three replicates.

## Results

### Identification and analysis of CDC14 in *A. flavus*

**Figure 1:**
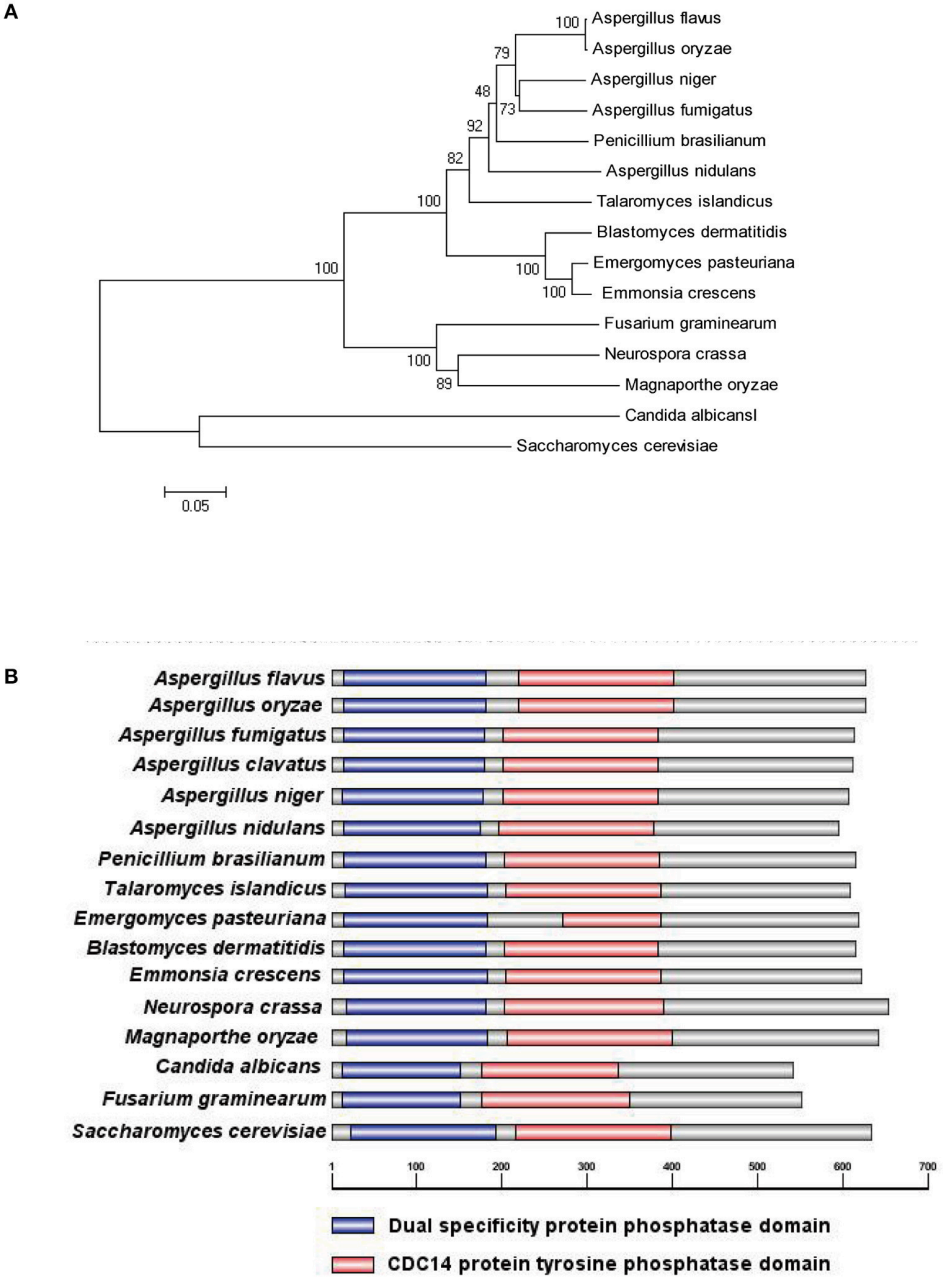
Identification of phosphatase CDC14 in *A. flavus*. **(A)** Phylogenetic analysis of putative CDC14 phosphatase in different fungi. The phylogenetic tree was constructed by MEGA 5.0 software and the Neighbour-joining method with 1,000 replicate. **(B)** Domain structure analysis of putative CDC14 protein from different fungi. The dual specificity protein phosphatase domain is shown in blue, while the CDC14 protein tyrosine phosphatase domain is shown in red.

To characterize the ortholog of the *S. cerevisiae* CDC14 in *A. flavus*, the *S. cerevisiae* CDC14 protein (DAA12468.1) sequence was used as the search query of the Basic Local Alignment Search Tool (https://blast.ncbi.nlm.nih.gov/Blast.cgi) in the NCBI database. The putative CDC14 protein (EED55756.1) in *A. flavus* was predicted to encode a 626 amino-acid protein with 39% (the highest) identity to the yeast CDC14. CDC14 protein sequences from various fungi, such as *Aspergillus spp, N. crassa, M. oryzae, C. albicans, F. graminearum*, and *S. cerevisiae* were downloaded from the NCBI database, and phylogenetic analysis was performed using the downloaded sequences (Figure 1A). The phylogenetic tree constructed based on CDC14 amino acid sequences revealed that *A. flavus* CDC14 is 100% identical to its homolog in the important industrial fungi *A. oryzae*, and 92% identical to its homolog in the related model species *A. nidulans*. These results showed that the CDC14 was highly conserved in *Aspergillus spp*. InterPro (http://www.ebi.ac.uk/interpro/scan.html) and IBS 1.0 software were used in protein domain analysis (Figure 1B), and the comparison results indicated that CDC14 protein phosphatase domain was conserved in fungi.

### Construction of the deletion (Δ*CDC14*) and complementation (Δ*CDC14*-Com) mutant strains

**Figure 2:**
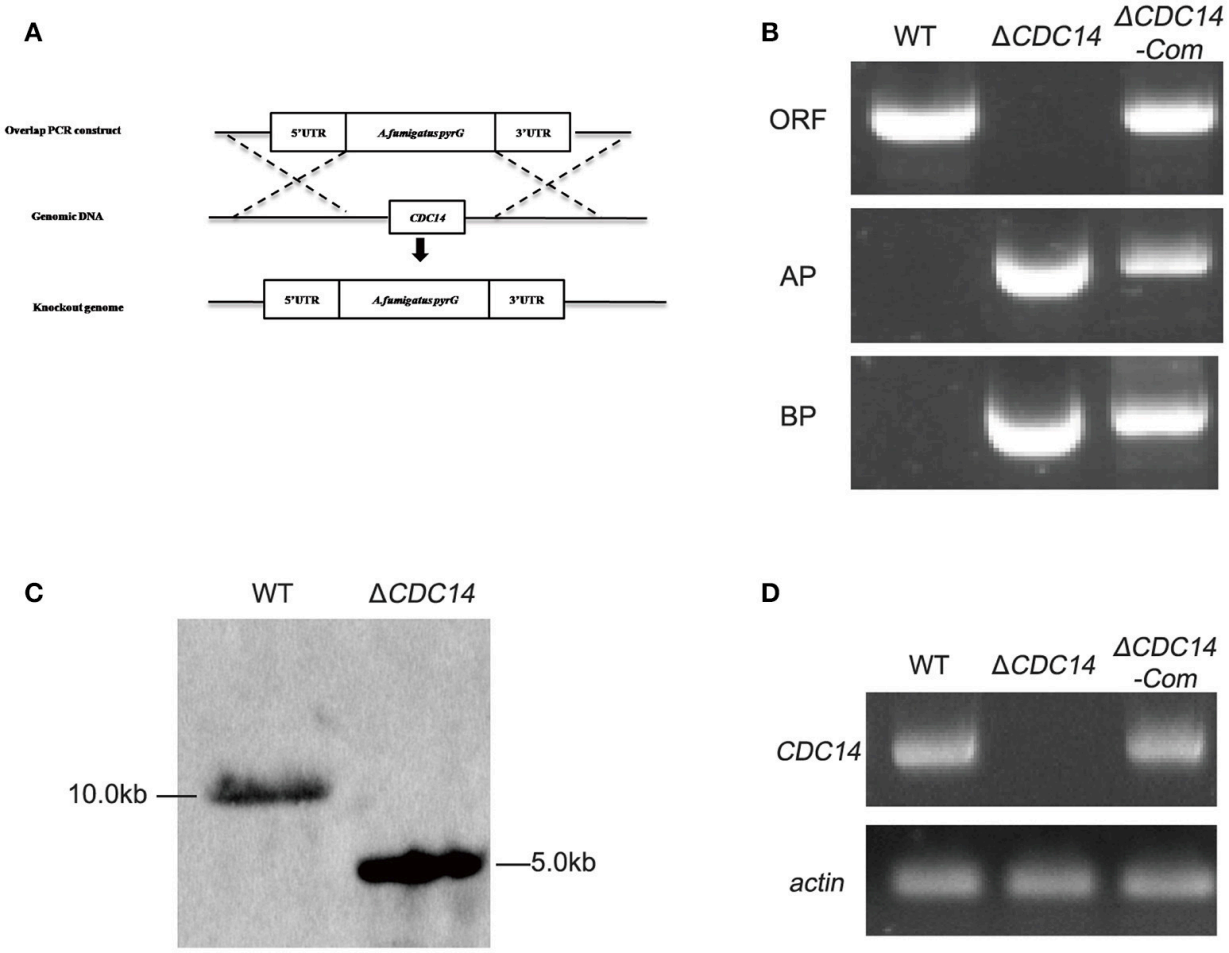
Construction of the *AflCDC14* deleted (Δ*CDC14*) and complemented (Δ*CDC14*-Com) strains. **(A)** Deletion strategy for Δ*CDC14* by using homologous recombination. **(B)** Δ*CDC14* and Δ*CDC14*-Com strains were verified by PCR analysis with genomic DNA as template. **(C)** Southern blot analysis of WT and Δ*CDC14* strains. Genomic DNA from each strain was digested with *Hind* III and hybridized with a 1.0 kb probe of the upstream region fragment of *AflCDC14* gene. **(D)** RT-PCR was used to detect the transcript levels of *AflCDC14* in different strains, and *actin* was used as the endogenous standard.

Homologous recombination strategy as shown in Figure 2A was used to generate Δ*CDC14* mutant. To ensure that the deletion of *AflCDC14* was directly responsible for the phenotype changes, Δ*CDC14* complementation strain (Δ*CDC14*-Com) was constructed by transforming the recombinant pPTR-*CDC14* plasmid into protoplasts of the *A. flavus* Δ*CDC14* strain. Transformants were confirmed by PCR (Figure 2B), Southern blot analysis (Figure 2C) and RT-PCR (Figure 2D). Southern blot analysis showed that the Δ*CDC14* mutant was successfully constructed, and the RT-PCR results indicated that the transcripts of *AflCDC14* were not detected in Δ*CDC14* strain in comparison to the WT and Δ*CDC14-*Com strains. As a result, *CDC14* deletionand complementation strains, donated as the Δ*CDC14* and Δ*CDC14-*Com were successfully obtained.

### *AflCDC14* is involved in vegetative growth

**Figure 3:**
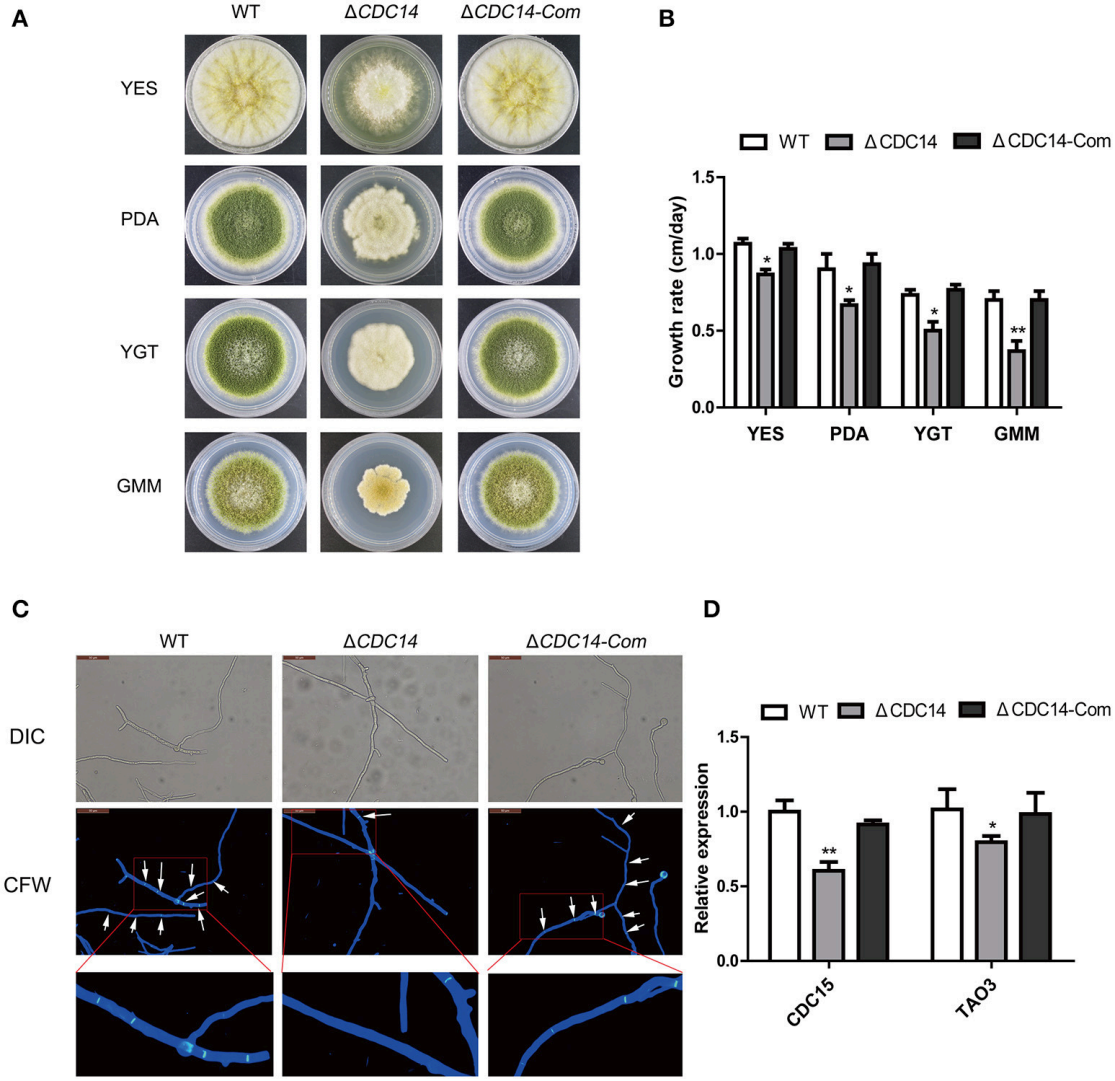
Growth and septum formation defects of the Δ*CDC14* mutant. **(A)** Colony morphology of WT, Δ*CDC14* and Δ*CDC14-*Com strains, grown on YES at 37°C for 3 d, and PDA, YGT, GMM media for 7 d. **(B)** Growth rate of WT, Δ*CDC14* and Δ*CDC14-*Com strains on different media. **(C)** Fluorescent images of hyphal cells stained with calcofluor white (CFW) revealed the defect of septum formation in Δ*CDC14* mutant, bars = 50 μm. **(D)** Relative transcript levels of septum formation related genes *CDC15* and *TAO3* were down-regulated, after cultured on PDA medium at 37°C for 48 h. *Actin* was used as the endogenous gene, and calculated by 2^−ΔΔCt^ method (*p ≤ 0.05, **p ≤ 0.01).

Colony morphology analyses revealed that the Δ*CDC14* mutant grew slowly compared to WT strain in YES, PDA, YGT, and GMM media (Figure 3A), but the growth defects of Δ*CDC14* were restored in the complemented strain Δ*CDC14-*Com (Figure 3A). Although the growth rate of Δ*CDC14* were generally reduced in comparison with the WT, it was more significant on low-nutrient media, YGT (32%) and GMM (49%), while less reduction was observed on PDA (25%) and nutrient-rich medium YES (16%) (Figure 3B). Altogether, these results suggested that *AflCDC14* is likely involved in vegetative growth. Previous studies have shown that CDC14 is necessary for cell septation in plant-pathogenic fungi *F. graminearum* (Li et al., 2015) and *M. oryzae* (Li et al., 2016). To confirm whether deletion of *AflCDC14* affects cell septation in *A. flavus*, we observed the septum formation of vegetative growth in all strains. Light microscopy observations showed that there were three to four septa in hyphae of WT and Δ*CDC14-*Com strains (Figure 3C, septa indicated by white arrow), but only one septum was observed in the Δ*CDC14* mutant (Figure 3C). The abnormal septum behavior in Δ*CDC14* mutant was well supported by the down-regulation of septum formation related genes *CDC15* and *TAO3* (Figure 3D). All these results indicated the importance of *AflCDC14* in the vegetative growth of *A. flavus*.

### *AflCDC14* is important for conidiogenesis

**Figure 4:**
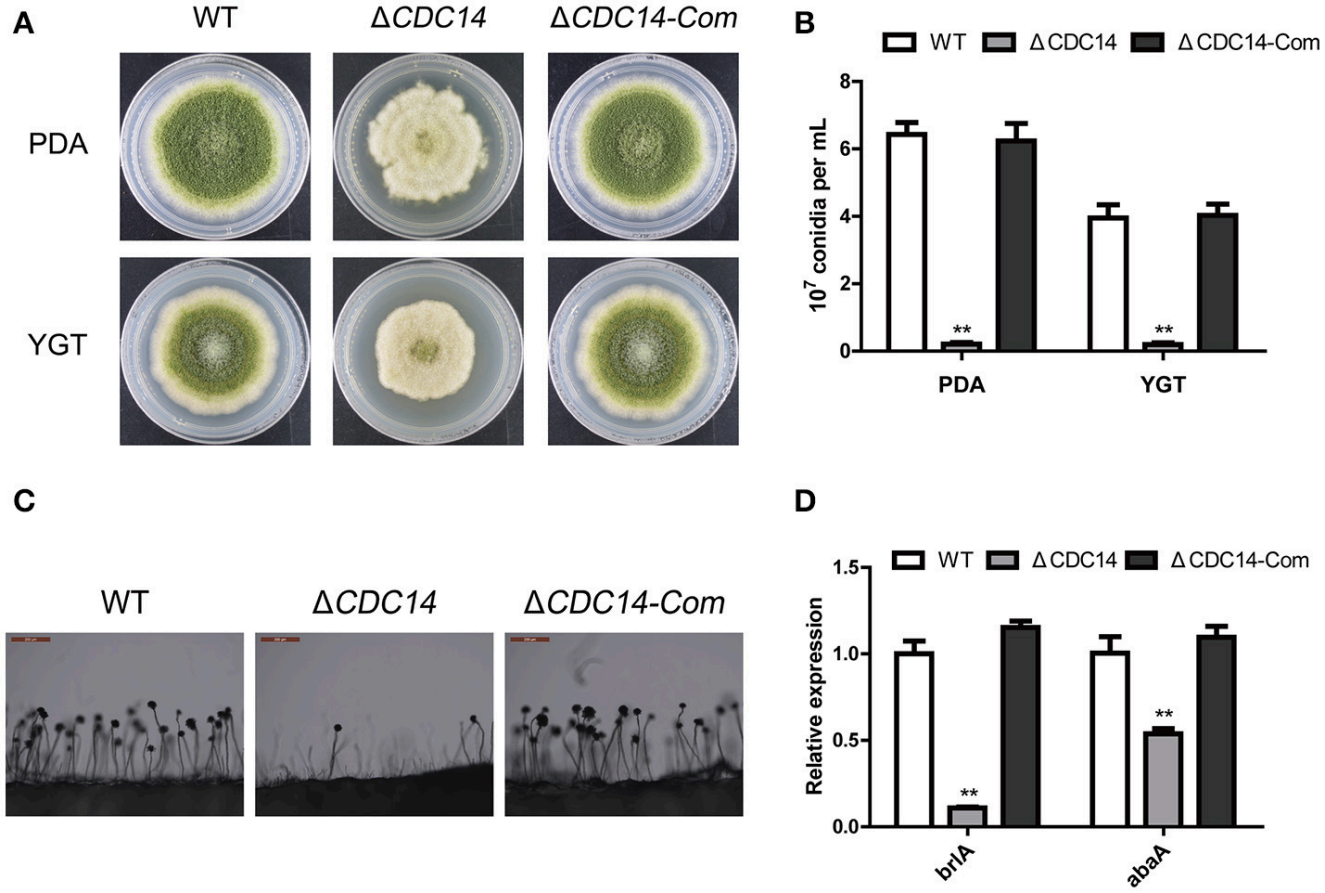
Deletion of *AflCDC14* caused defects of conidiation in *A. flavus*. **(A)** Colonies of WT, Δ*CDC14* and Δ*CDC14-*Com strains cultured on PDA and YGT media at 37°C for 5 d. **(B)** Conidia amount produced by the different strains on PDA and YGT media. **(C)** Conidiophores morphology of WT and *CDC14* mutants observed by light microscope after 12 h incubation, bars = 200 μm. **(D)** Expression levels of conidia-related genes *brlA* and *abaA* of all the strains after 48 h incubation (**p ≤ 0.01).

To investigate the bio-function of *AflCDC14* gene in conidiation, PDA and YGT medium were inoculated with the strains (WT, Δ*CDC14* and Δ*CDC14-*Com) and then cultured at 37°C in the dark. After 5 days, Δ*CDC14* exhibited a significant decrease in conidiation compared to WT and Δ*CDC14-*Com strains (Figure 4A). The number of conidia produced by the Δ*CDC14* mutant on PDA and YGT plates was reduced more than 10-fold compared to the WT (Figure 4B). Microscopic examination revealed that the Δ*CDC14* mutant formed lower number of conidiophores (Figure 4C). To gain further insight into the role of *AflCDC14* in conidiation, qRT-PCR was performed to detect the transcript levels of two conidia-related genes *brlA* and *abaA*, and the results showed that the expression levels of these two genes were both down-regulated in the Δ*CDC14* mutant compared to WT and Δ*CDC14-*Com strains (Figure 4D). These results indicated that *AflCDC14* plays a critical role in the conidiation of *A. flavus*.

### *AflCDC14* is essential for sclerotial formation

**Figure 5:**
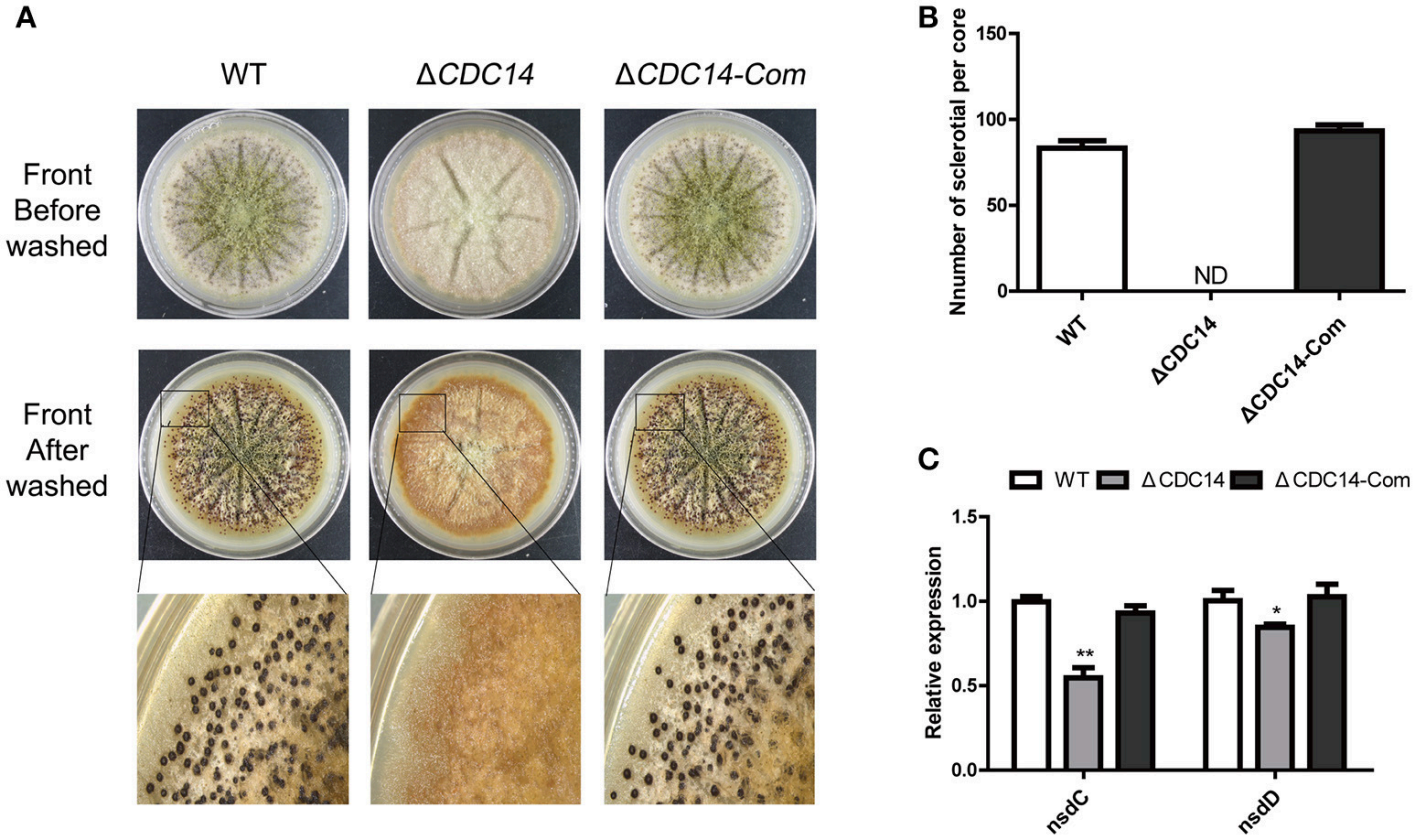
*AflCDC14* was essential for sclerotial formation. **(A)** Phenotypic characterization of WT, Δ*CDC14* and Δ*CDC14-*Com strains on Wickerham medium at 37°C for 7 days. **(B)** Sclerotial amounts produced by *A. flavus* strains. ND means not be defined. **(C)** Expression levels of sclerotial related genes *nsdC* and *nsdD* in different strains for 48 h (*p ≤ 0.05, **p ≤ 0.01).

In order to resist unsuitable environment, a structure of sclerotial is formed in *A. flavus*. After being cultured on Wickerham (WKM) medium for 7 days at 37°C in the dark, 70% ethanol was used to wash off aerial hyphae and conidia, and the result showed that sclerotia production was completely impaired in Δ*CDC14*, in contrast to the WT and Δ*CDC14-Com* strains (Figures 5A,B). Furthermore, a quantification of the expression levels of genes *nsdC* and *nsdD*, which influence sclerotia formation, showed a decrease in Δ*CDC14* compared to WT and Δ*CDC14-Com* strains (Figure 5C). These results suggested that *AflCDC14* is essential for sclerotia formation in *A. flavus*.

### *AflCDC14* may play a negative role in regulating aflatoxin biosynthesis

**Figure 6:**
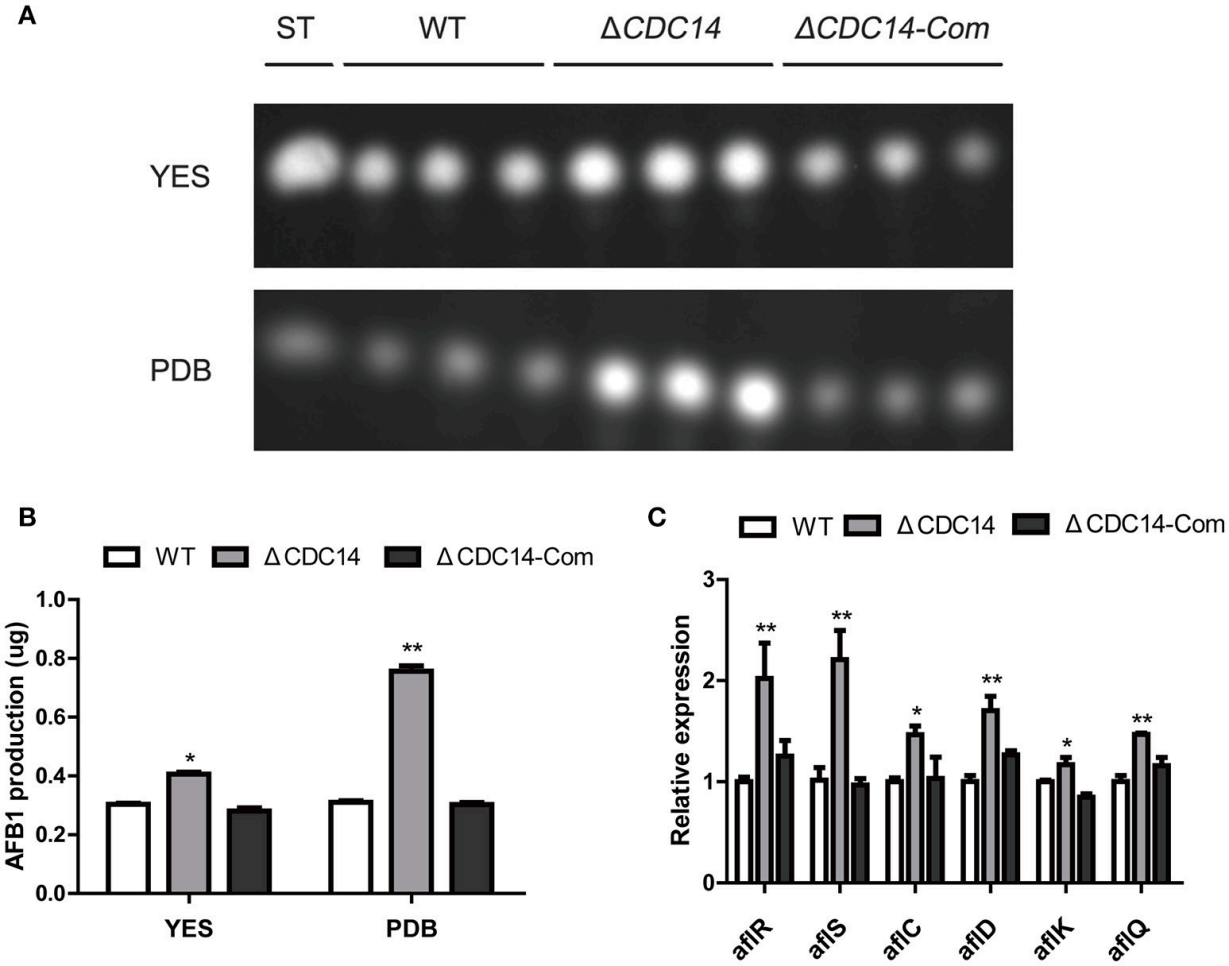
Aflatoxin production of the WT, Δ*CDC14* and Δ*CDC14-*Com strains. **(A)** TLC assay of AFB1 production by the WT, Δ*CDC14* and Δ*CDC14-*Com strains in YES and PDB liquid media cultured at 29°C for 6 days. ST indicates AFB1 standard. **(B)** Quantification analysis of AFB1 as in **(A)**. **(C)** Relative transcript levels of six aflatoxin biosynthesis-related genes in different strains for 48 h. *Actin* was used as the endogenous gene, and calculated by 2^−ΔΔCt^ method (*p ≤ 0.05, **p ≤ 0.01).

In our above described experiments, we found that *AflCDC14* may be involved in the secondary metabolism of *A. flavus* (Figure 5A). Thus, we investigated the effect of *AflCDC14* on aflatoxin production, which is the most crucial and toxic secondary metabolite in *A. flavus*. TLC assay and quantitative analysis showed a significantly increased aflatoxin production in Δ*CDC14* compared to WT and Δ*CDC14-*Com strains when cultured in both YES liquid medium and PDB medium (Figures 6A,B). To examine the effect in more detail, qRT-PCR was performed to analyze the transcript levels of the aflatoxin biosynthesis-related genes. Consistent with the TLC results, the expression levels of aflatoxin-specific regulatory genes (*aflR, aflS*), early-expressed structural genes (*aflC, aflD*), mid- and late-expressed genes (*aflK* and *aflQ*) in Δ*CDC14* mutant were all higher than those of the WT and Δ*CDC14-*Com strains (Figure 6C). Taken together, all these results demonstrated that *AflCDC14* may play a negative role in aflatoxin biosynthesis in *A. flavus*.

### *AflCDC14* response to multiple stresses in *A. flavus*

**Figure 7:**
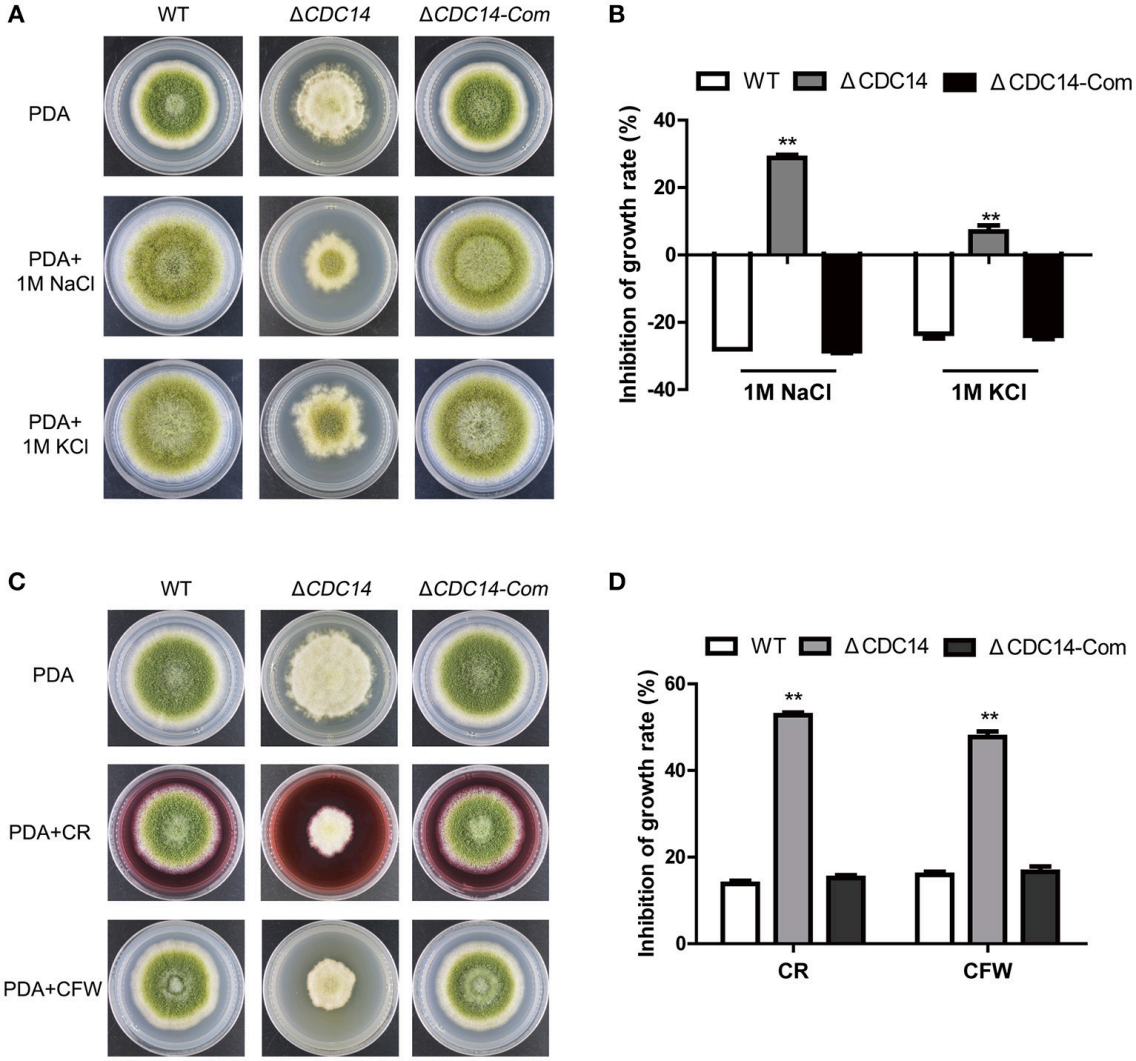
The *AflCDC14* is involved in response to hyperosmotic and cell wall integrity stresses. **(A)** Colonies of WT, Δ*CDC14* and Δ*CDC14-*Com strains on PDA media with 1 M NaCl and 1 M KCl for 4 days. **(B)** Growth inhibition of each strain under hyperosmotic stress. [inhibition of growth rate = (the diameter of untreated strain- the diameter of treated strain)/(the diameter of untreated strain) × 100%]. **(C)** Morphology of different strains were grown on PDA media supplemented with 200 μg/mL CFW and 200 μg/mL CR for 5 days. **(D)** The inhibition growth rate of WT, Δ*CDC14* and Δ*CDC14-*Com strains under cell wall integrity stress (**p ≤ 0.01).

Previous studies have shown that CDC14 participated in multi-stresses response in fungi *S. cerevisiae* (Bodenmiller et al., 2010) and *B. bassiana* (Wang et al., 2013). Therefore, we were interested in exploring the role of *AflCDC14* in response to various stress agents. Relative growth inhibition was used as a standard for measuring stress response. As shown in Figures 7A,B, the relative growth inhibition of Δ*CDC14* induced by osmotic stress agents (1 M NaCl and 1 M KCl) was significantly higher compared to WT and Δ*CDC14-*Com strains, suggesting that the Δ*CDC14* mutant was more sensitive to the hyperosmotic stress than the other two strains. Similarly, Δ*CDC14* mutant also exhibited increased susceptibility to the cell wall integrity agents CR and CFW (Figures 7C,D). Whereas, there was no growth inhibition by the addition of H_2_O_2_ (Oxidative stress) and MMS (Genotoxic stress) agents (Data not shown). These findings indicated that *AflCDC14* is involved in response to high osmotic and cell wall integrity stresses in *A. flavus*.

### *AflCDC14* contributes to pathogenicity in crop seeds

**Figure 8:**
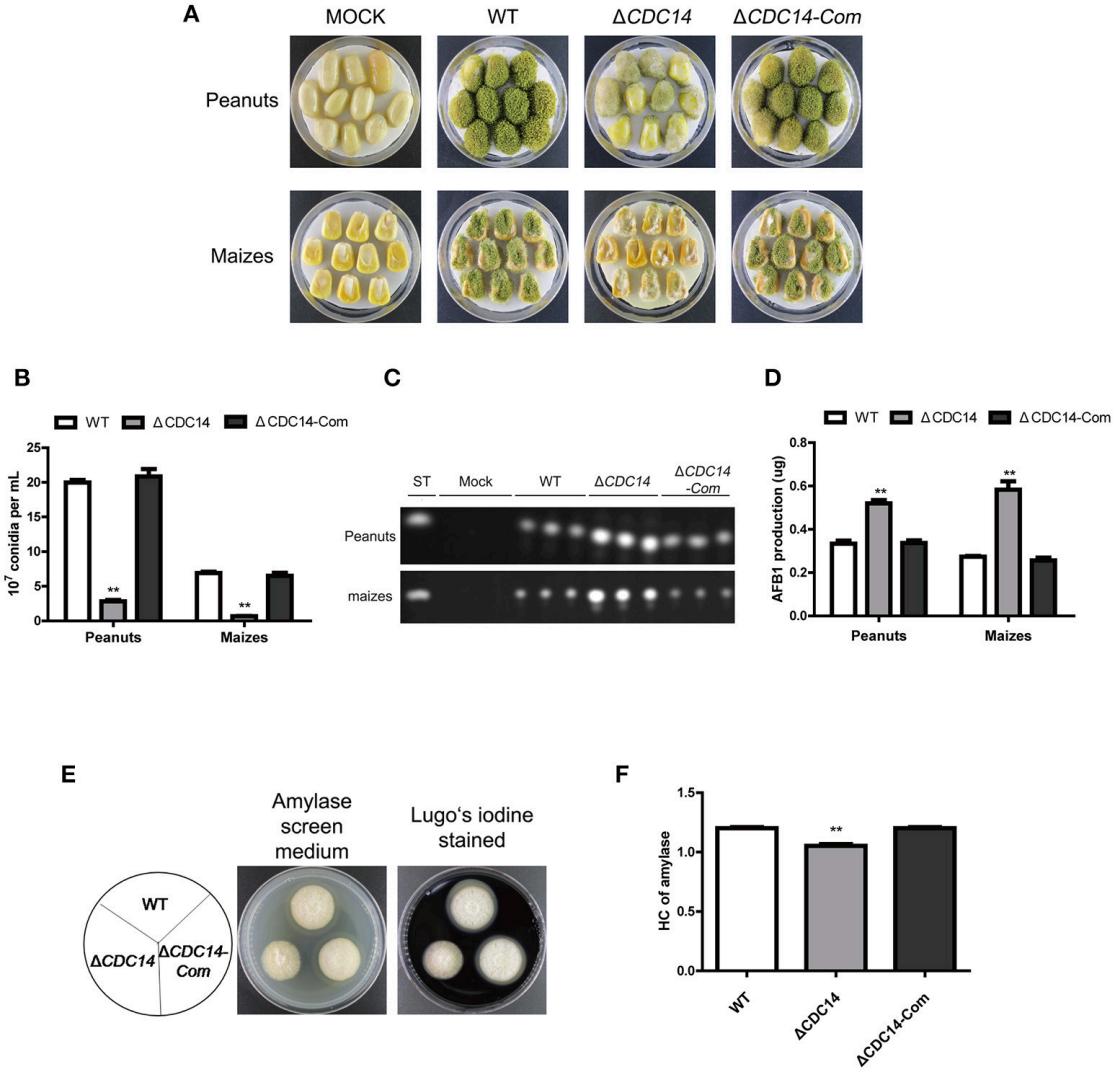
Δ*CDC14* mutant was impaired in the infection of plant seeds. **(A)** Peanut and maize seeds were inoculated with WT, Δ*CDC14* and Δ*CDC14-*Com strains and cultured at 29°C for 6 days. **(B)** Quantification analysis of conidia from the infected peanut and maize seeds. **(C)** TLC was used to detect the AFB1 production extracted from the infected peanut and maize seeds. **(D)** Quantification of AFB1 production as in **(C)**. **(E)** Assay of lipase activity in different strains and the amylase screening medium was stained with Lugo's iodine. **(F)** Quantification analysis of amylase's HC according to the results of **(E)**. HC = the diameter of transparent zonel/the diameter of colonies (**p ≤ 0.01).

Based on previous results of Δ*CDC14* exhibiting a variety of defects invegetative growth, conidiation and sclerotia formation, we proposed that *AflCDC14* might play roles in the infection of crop seeds by *A. flavus*. The importance of *AflCDC14* to *A. flavus* pathogenicity was evaluated by inoculation of peanut and maize seeds with conidial suspension from WT, Δ*CDC14* and Δ*CDC14-*Com strains. After 5 days of inoculation, WT and Δ*CDC14-*Com infection resulted in full virulence on all peanut and maize seeds, while Δ*CDC14* mutant was severely impaired in the colonization of peanut and maize seeds (Figure 8A). Then we measured conidial production in these infected seeds, and the deletion of *AflCDC14* resulted in a significant reduction in conidial production compared to WT and Δ*CDC14-*Com mutant (Figure 8B). We also assayed the amount of aflatoxin produced on infected seeds, and TLC assays showed that the Δ*CDC14* mutant produced more aflatoxin on peanut and maize seeds than WT and Δ*CDC14-*Com strains (Figures 8C,D), consistent with the prior results of aflatoxin biosynthesis of Δ*CDC14* mutant in YES and PDB liquid media. As amylase was considered to be associated with pathogenicity in *Aspergillus spp*. (Alam and Kelly, 2017), we detected the activity of amylase in the different strains, and the results showed that the activity of amylase was significantly decreased in Δ*CDC14* compared to WT and Δ*CDC14-*Com strains (Figures 8E,F). All these data illustrated that *AflCDC14* of *A. flavus* is important for crop seeds pathogenicity.

### Subcellular localization of AflCDC14

**Figure 9:**
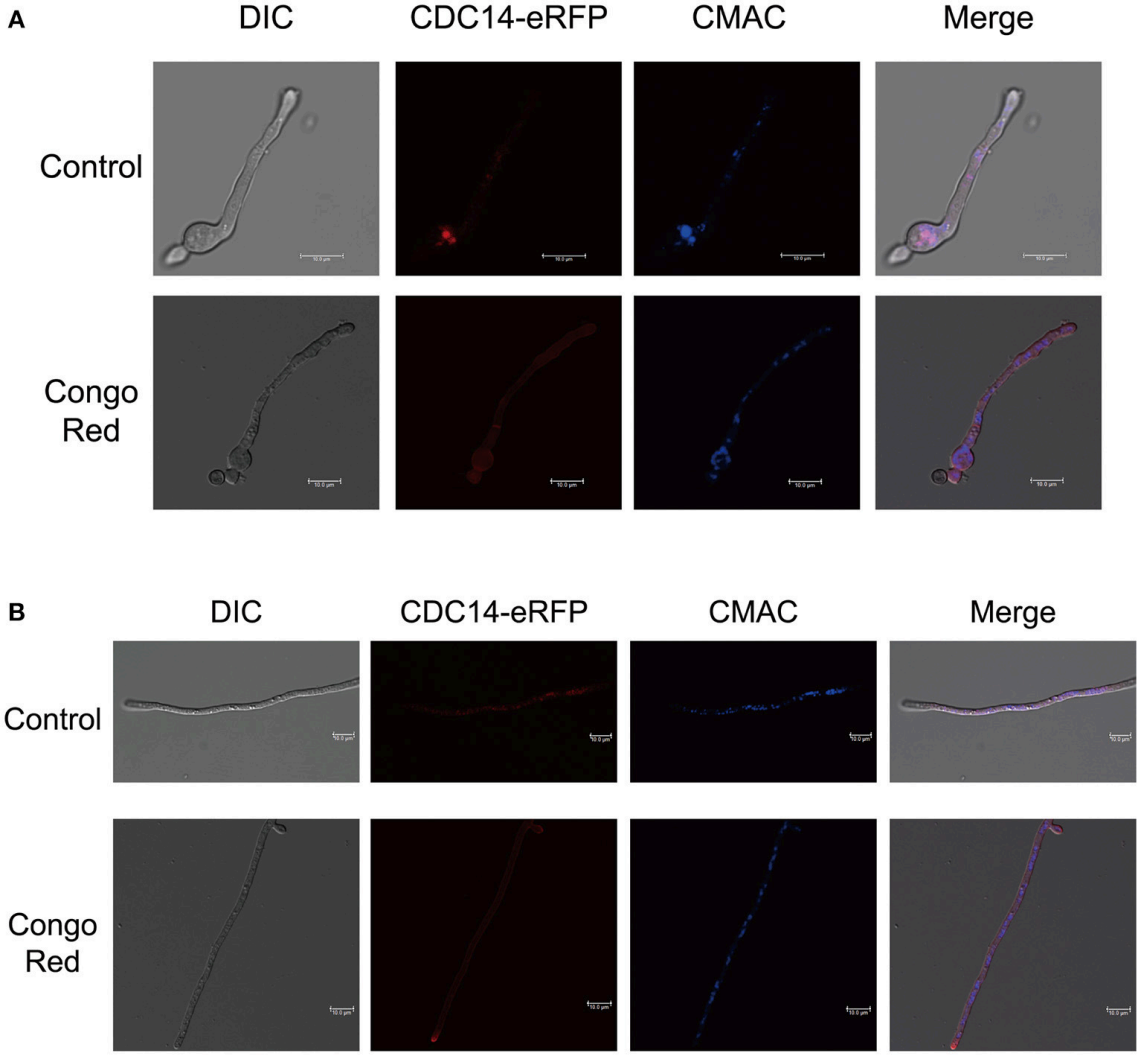
Subcellular localization of AflCDC14-eRFP in *A. flavus*. **(A)** Fluorescent image of CDC14-eRFP during the conidia germination period. The CDC14-eRFP strain was grown for 12 h at 37°C in PDB medium and transferred to congo red for 0.5 h. The vesicles was stained by chloromethyl derivative of aminocoumarin (CMAC). bars = 10 μm. **(B)** Localization of CDC14-eRFP in hyphal stages. The CDC14-eRFP strain was grown for 12 h at 37°C in PDB medium and transferred to congo red for 0.5 h. bars = 10 μm.

For subcellular localization assays, a *CDC14*-eRFP fusion construct with its native promoter was generated and transformed into protoplasts of *A. flavus CA14 PTS* strain. The resulting transformant exhibited a similar phenotype as WT strain, indicating that the eRFP-tag had no impact on the CDC14 function (data not shown). When examined for its subcellular localization in conidia germination stage, eRFP signals were mainly observed in cytoplasm and vesicles by staining with CMAC (Figure 9A), and most CDC14 protein stored in the head of spore. Similarly, as shown in Figure 9B, we discovered that eRFP signals were present in both the cytoplasm and vesicles of the hyphae. Our previous results showed that *AflCDC14* is involved in response to high osmotic and cell wall integrity stresses in *A. flavus*. Hence, we observed the subcellular localization of CDC14-eRFP strain under stress conditions. We have not observed any difference in the CDC14-eRFP localization in the presence of agent NaCl (data not shown). However, after being treated with CR for 0.5 h, we discovered that eRFP signals were enriched in all cytoplasm rather than vesicles (Figures 9A,B). These results indicated that AflCDC14 is mainly localized to the cytoplasm and vesicles, and with greater enrichment in the cytoplasm under cell wall integrity stress condition.

## Discussion

Reversible phosphorylation and dephosphorylation, catalyzed by kinases and phosphatases, respectively, regulate various cellular processes, including cell cycle, signal transduction and secondary metabolism in fungi (Breitkreutz et al., 2010; Wurzenberger and Gerlich, 2011). Previous studies have demonstrated that phosphorylation plays a critical role in the regulation of asexual development and aflatoxin production in *A. flavus* (Ren et al., 2016). CDC14 is well conserved in diverse fungi for regulation of mitosis and cytokinesis by dephosphorylating CDKs in phosphorylation sites (Chen et al., 2008; Bloom et al., 2011). However, studies on CDC14 in *Aspergillus spp*. are still rare. Thus, we found it worthwhile to characterize the function of CDC14 phosphatse in *A. flavus*. In this study, our results indicated that CDC14 is important for asexual development, secondary metabolism and pathogenicity of *A. flavus*.

The *A. flavus CDC14* gene is an ortholog of the *F. graminearum* (Li et al., 2015) and *M. oryzae* (Li et al., 2016) *CDC14* genes, both of which have been proved to be involved in asexual and sexual development. Here, we found that the growth rate of the Δ*AflCDC14* mutant was significantly reduced (Figure 3), which is similar to the Δ*CDC14* mutant in *M. oryzae* (Li et al., 2016) and *B. bassiana* (Wang et al., 2013). However, when the *CDC14* ortholog was knocked out in *A. nidulans* (Son and Osmani, 2009), there was no distinct defect in growth rate. Given that appropriate cell cycle regulation is important for fungal development in yeast and other filamentous fungi, we speculated that the deletion of *AflCDC14* may affect cytokinesis in vegetative hyphae, which is consistent with the phenotype defects of septum and down-regulation of septum formation related genes *CDC15* (Fankhauser and Simanis, 1993) and *TAO3* (Gupta et al., 2016) in Δ*CDC14* mutant. Our study also showed that deletion of *AflCDC14* resulted in a severely defective conidia production and morphology (Figure 4). The abnormal conidiation in Δ*CDC14* mutant is well supported by the serious down-regulation of the expression of conidia-related transcription factors *brlA* and *abaA* (Tao and Yu, 2011) in *A. flavus*. Besides reduction in vegetative growth and conidiation, Δ*CDC14* failed to produce sclerotia (Figure 5), which are considered to be derived from sexual structures cleistothecia to adapt to unfavorable environment, indicating that *AflCDC14* contributes to *A. flavus* sexual development. In plant pathogenic fungi *F. graminearum* (Li et al., 2015) and *M. oryzae* (Li et al., 2016), deletion of *CDC14* led to a specific defect in sexual development. We also found that the expression of the sexual development related genes *nsdC* and *nsdD* (Cary et al., 2012), were reduced in the Δ*CDC14* mutant. Therefore, these results indicated that *AflCDC14* may play a critical role in regulating asexual and sexual development in *A. flavus*.

Mitogen-activated protein kinases (MAPK) cascades are highly conserved eukaryotic signal transduction systems in almost all eukaryotes. The MAPK cascades have been identified in several filamentous fungi, including *Fusarium spp*. (Zheng et al., 2012), *Aspergillus spp*. (Vito et al., 2015), *M. oryzae* (Jin et al., 2013), and *B. cinerea* (Heller et al., 2012). In *A. nidulans*, three MAPK pathways (fus3/kss1-MAPK, Hog1-MAPK, slt2-MAPK) have been characterized to be involved in response to multi-stress, including nutrient, hyperosmotic and cell wall integrity signaling, respectively, indicating that proper phosphorylation of MAPK pathways play an important role in multi-stress response (Bayram et al., 2012). As members of phosphatases, orthologs of *AflCDC14* in various fungi participated in multi-stress response via dephosphorylation regulation. In our study, Δ*CDC14* displayed increased susceptibility to osmotic and cell wall integrity stresses in *A. flavus* (Figure 7). In *B. bassiana*, the Δ*CDC14* mutant was sensitive to oxidative, osmotic and cell wall stresses, which have been found to be associated with the MAPK related high osmotic (HOG) and cell wall integrity (CWI) pathways (Wang et al., 2013, 2016). Similarily, the ortholog of *AflCDC14* in *S. cerevisiae*, CDC14 is also a core phosphatase in the signaling network by regulating response to various stresses (Breitkreutz et al., 2010). However, *AflCDC14* ortholog in *A. fumigatus*, CDC14 does not interfere with osmotic stress response but is involved in response to cell wall integrity stress agents (Winkelströter et al., 2015b). These observations imply that CDC14 may regulate multi-stress response in a species-specific manner. It seems that CDC14 may be related to cross-talking among HOG and CWI-MAPK pathways, which is critical for signal transduction under various stress conditions. The exact mechanism is required to investigate the relationship between CDC14 and multi-stress response.

Although the biosynthesis pathway of aflatoxin has been well characterized, the regulatory mechanism is complicated and has not been fully understood. Previous studies have revealed that this pathway may be affected by various elements, including protein post-translation modifications such as phosphorylation (Ren et al., 2016), acetylation (Lan et al., 2016), methylation (Li et al., 2017), SUMOylation (Nie et al., 2016), and some environmental factors (Zhang et al., 2015) in *A. flavus*. It was demonstrated in the study that the aflatoxin production by Δ*CDC14* was higher than those of WT and Δ*CDC14-*Com strains (Figure 6), which corresponds with the up-regulation of aflatoxin biosynthesis regulation genes *aflR, aflS*, and aflatoxin biosynthesis structural genes *aflC, aflD, aflK*, and *aflQ*. We conclude that deletion of *AflCDC14* may alter the phosphorylation level of its CDK and MAPK substrates, which are important for secondary metabolism in filamentous fungi (Bayram et al., 2012; Liu et al., 2015). On the other hand, it is possible that inactivation of *AflCDC14* may lead to the alteration of post-translation modification of regulation genes *aflR* and *aflS*. Taken together, these data suggested that *AflCDC14* may play a crucial role in secondary metabolism in *A. flavus*.

It is well-known that phosphatases display critical roles in the pathogenesis of pathogenic fungi, including *M. oryzae* (Liu et al., 2016)*, C. albicans* (Lee et al., 2004) and *A. fumigatus* (Winkelströter et al., 2015a). To investigate the bio-function of *AflCDC14* in *A. flavus* pathogenicity, we observed seeds infection in the Δ*CDC14* mutant, and the result showed that deletion of *AflCDC14* led to defective colonization of both peanut and maize seeds (Figure 8). This finding is similar to studies on the deletion of *AflCDC14* ortholog in *M. oryzae* (Li et al., 2016) and *C. albicans* (Clemente-Blanco et al., 2006). One contributing factor to this defect in seeds infection could be related to the inhibition in growth and conidiation. We also discovered that the activity of amylase in Δ*CDC14* was lower compared to WT and Δ*CDC14-Com* strains. Amylase was considered to be associated with pathogenicity in *Aspergillus spp*. (Alam and Kelly, 2017; Li et al., 2017). Therefore, it is likely that the lower amylase activities may contribute to reduced virulence in the Δ*CDC14* mutant. All these evidences highlight that phosphatase CDC14 may be critical for pathogenicity in *A. flavus*.

Interestingly, our results indicated that *A. flavus* AflCDC14 mainly localized to the cytoplasm and vesicles during coidial germination and mycelial development stages, which is different from its ortholog in *F. graminearum* (Li et al., 2015) and *M. oryzae* (Li et al., 2016). In plant-pathogenic fungi *F. graminearum* and *M. oryzae*, the ortholog of AflCDC14 were all localized to nucleus and spindle pole body (SPB). In human pathogenic fungi *C. albicans*, CaCDC14-YFP began to accumulate both in the nucluus and nucleolar, and then degraded (Clemente-Blanco et al., 2006). The difference of ortholog of CDC14 subcellular localization may be in a species-specific manner. Our previous results showed that *AflCDC14* is involved in response to high osmotic and cell wall integrity stresses in *A. flavus*. We found that after being treated with CR for 0.5 h, eRFP signals were enriched in all cytoplasm rather than vesicles (Figures 9A,B), which is different from control. This may be one of the reasons why *AflCDC14* respond to cell wall integrity stresses, and futher research need to investigate the exact mechanism on *AflCDC14* response to stresses.

In summary, the phosphatase CDC14 was identified in *A. flavus*, and we investigated the importance of CDC14 during growth, development and aflatoxin biosynthesis in *A. flavus*. Our findings suggest that CDC14 plays critical role in vegetative growth, conidiation, sclerotia formation and aflatoxin biosynthesis. Additionally, CDC14 also affect osmotic and cell wall integrity stresses response, and pathogenicity. To our knowledge, this is the first report on the function of phosphatase in *A. flavus*. However, further investigation is necessary to discover the molecular mechanism of phosphatase CDC14 in association with some important signal pathways.

## Author contributions

GY, ZZ, and SW conceived and designed the experiments. GY, YH, and LC performed the experiments. YY and YQ contributed reagents, materials and analysis tools. GY, OF, XW, and SW wrote and revised the paper. SW supported financially and gave final approval of manuscript.

### Conflict of interest statement

The authors declare that the research was conducted in the absence of any commercial or financial relationships that could be construed as a potential conflict of interest.
